# Vitamin D is not required for adaptive immunity to listeria

**DOI:** 10.14814/phy2.14209

**Published:** 2019-08-28

**Authors:** Gary A. Baisa, Lori Plum, Steve Marling, Jeremy Seeman, Hector F. DeLuca

**Affiliations:** ^1^ Department of Biochemistry University of Wisconsin‐Madison Madison Wisconsin; ^2^ Diasorin Inc. Stillwater Minnesota

**Keywords:** Vitamin D

## Abstract

Although *ex vivo* research suggests that vitamin D may play a role in innate and adaptive immunity, clear *in vivo* evidence is lacking. We have tested whether severe vitamin D deficiency alters the ability of mice to resist infection by Listeria. Our results show that vitamin D deficiency does not affect the LD_50_ of naïve mice in response to Listeria. To study the adaptive immune response, the LD_50_ for Listeria‐immunized mice was determined for vitamin D‐deficient and vitamin D‐sufficient mice. Although the LD_50_ clearly increased by immunization with inactivated Listeria, there was no effect of vitamin D deficiency on survival of mice infected with wild‐type Listeria. Thus, in this model of adaptive immunity, we could find no evidence of a role for vitamin D.

## Introduction

Observational studies have long suggested a relationship between low vitamin D levels and an increase in susceptibility to infectious diseases. One of the first infectious diseases associated with low vitamin D levels and an increase in disease prevalence was *Mycobacterium tuberculosis* infections (Nnoaham and Clarke, 2008; Huang et al., 2017). Interestingly, cod liver oil, which is high in vitamin D, was used as an early treatment of *M. tuberculosis* infections (Green, 2011; Williams, 1849). Lower vitamin D levels are also associated with an increased prevalence of respiratory (Berry et al., 2011; Ginde et al., 2009) and fungal infections (Lim et al., 2015), and an increased risk of sepsis (de Haan et al., 2014; Upala et al., 2015).

Although vitamin D and its active metabolite, 1,25 hydroxyvitamin D_3_ [1,25(OH)D_3_], show an effect on immune cell development and activity in the innate and adaptive immune systems, an impact on disease outcomes has not been determined (recently reviewed (Lang and Aspinall, 2017)). Mouse models of vitamin D deficiency and infectious disease have been combined to investigate a possible role of vitamin D in the immune response to an infectious disease. Bruce et al. showed that *Listeria monocytogenes*‐infected vitamin D receptor knockout (VDR‐KO) mice had a slower bacterial clearance rate when compared to wild‐type mice. However, the VDR‐KO mice ultimately cleared the infection and no difference in disease outcome was observed (Bruce et al., 2009). Ehrchen et al. utilized *Leishmania major* to infect VDR‐KO mice to show these mice cleared the infection faster than the wild‐type strain but showed no difference in disease outcome (Ehrchen et al., 2007). In a study utilizing a mouse model of urinary tract infections (UTI), vitamin D‐deficient mice had a higher bacterial burden in their kidneys when compared to vitamin D‐sufficient mice but the effect on disease outcome was not investigated (Hertting et al., 2017). Vitamin D‐deficient mice infected with *Mycobacterium bovis* had higher bacterial burdens in their lungs when compared to a control population but again this study did not analyze final disease outcome (Yang et al., 2013).

In the present study, the ability of proven vitamin D‐deficient mice to defend against *Listeria monocytogenes* infection has been compared to that of vitamin D‐sufficient mice. These studies were carried out in both naïve mice and mice immunized with attenuated *Listeria*.

## Materials and Methods

### Animal husbandry

Female C57Bl/6J weanling (3‐week‐old) mice were purchased from Jackson Laboratory (Bar Harbor, ME) and housed in the University of Wisconsin–Madison Department of Biochemistry vivarium. Mice were placed in high density cages at a maximum of 5 mice/cage containing corncob bedding, half a square of mouse nestlet, and a mouse enrichment igloo. Cages were changed biweekly with fresh diet provided. All lighting (fluorescent) was covered with filters to eliminate production of vitamin D. Mice were exposed to 12 h light–dark cycles. All procedures were approved by the Research Animal Resources Committee of the College of Agricultural & Life Sciences, University of Wisconsin–Madison.

### Diets

Diet and RO water were provided *ad libitum*. Mice were fed an in‐house formulated diet, Diet 11, containing calcium concentrations of 0.02%, 0.47%, or 2% (Yang et al., 1993). These diets were provided with and without vitamin D supplementation. The Diet 11 2% calcium diet also contained 10% lactose to aid in calcium absorption. All diets were stored at 4°C with a 3‐month expiration date.

### Induction of vitamin D deficiency by dietary calcium cycling method

To deplete vitamin D stores, 3‐week‐old weanling mice were reared for 1 week on Diet 11 0.47% calcium (study week 1), 3 weeks on Diet 11 0.02% calcium (study weeks 2–4), 1 week on Diet 11 0.47% calcium (study week 5), and then 3 additional weeks on Diet 11 0.02% calcium (study weeks 6–8). Serum calcium levels were measured at the end of study week 8. If the average serum calcium levels were >6.5 mg/dL, mice were kept on the Diet 11 0.02% calcium diet for an additional week and serum calcium levels were again determined. This process was continued until the average serum calcium levels were <6.5 mg/dL. Mice were then placed on the Diet 11 10% lactose 2% calcium rescue diet until the average serum calcium levels of ≥9.0 mg/dL were achieved (~4–5 weeks). The mice were then maintained on the Diet 11 10% lactose 2% calcium diet up to and through the infection experiments.

As a final confirmation of vitamin D deficiency, pooled serum samples were assayed for 25(OH)D_3_ concentrations. Once serum 25(OH)D_3_ levels were confirmed to be below the limits of detection (<4 ng/mL), the mice were used in the studies.

Vitamin D‐sufficient control mice were placed on the same dietary calcium cycling schedule as the vitamin D‐deficient mice but their diets were supplemented with 7 µg of vitamin D_3_ per week.

### Blood collection and processing

All blood draws were performed by maxillary bleed and collected directly into a 1.5 mL tube. Blood was allowed to coagulate for a minimum of 30 min and then centrifuged at low speed (1500*g*) for 15 min. Serum was transferred to a new 1.5 mL tube and centrifuged at high speed (15,000*g*) for 1 min. The serum was then transferred to a new 1.5 mL tube and stored at −20°C.

### Serum calcium and 25(OH)D_3_ measurements

Serum calcium levels were determined by atomic absorption spectroscopy. Individual mouse serum samples were diluted (1:40) in 0.1% lanthanum chloride prior to analysis on a Perkin Elmer 900H Atomic Absorption Spectrometer (Waltham, MA). Infections were not initiated until serum calcium levels in the D‐deficient mice returned to normal (as compared to vitamin D‐sufficient mice). No difference in the serum calcium levels of the two groups was also determined statistically.

Pooled serum samples were assayed for 25(OH)D_3_ levels by an in‐house high‐performance liquid chromatography (HPLC)‐based method (Irving et al., 2015; Irving et al., 2018) or by DiaSorin (Stillwater, MN) using a chemiluminescent immunoassay (Liaison^®^ 25 OH Vitamin D Total Assay).

### *Listeria monocytogenes* culture and inoculum preparation

The *Listeria monocytogenes* wild‐type strain 10403S and the derivative vaccine strain Δ*actAΔinlB*, also referred to as the Listeria Attenuated Double Deletion (LADD) strain (Brockstedt et al., 2004), were grown on brain heart infusion media. For Listeria inoculum preparations, each strain was grown statically overnight at 30°C. The culture was then subcultured (1:4) into prewarmed BHI broth and grown at 37°C with shaking (250 RPM) to an early log phase of a Laxco DSM meter OD_600_ = 0.14–0.16 (3.32E+08 CFUs/mL). A calculated culture volume was then diluted into PBS to achieve the target CFUs/inoculation volume (200 µL). For the Listeria dose–response experiments, subsequent inoculum dilutions were made in PBS. Inocula were immediately placed on ice once prepared.

### Bacterial infection of mice

Prior to performing the intravenous tail vein infection procedure, the mice were warmed under a heat lamp for 1–2 min. The mice were then restrained and their tails washed with 70% ethanol. Using a 1 mL syringe with a 30 g needle, 200 µL of the Listeria/PBS suspension was administered through the tail vein.

### Dose–response and survival assays

The dose–response experiments used five dosing groups each containing 5–10 mice per group. An additional 3–5 mice were included as negative controls. Depending on the experiment, the Listeria dosage [colony forming units (CFUs)] between groups varied from 2–5‐fold. The negative controls were inoculated with diluent only (PBS). Once inoculated, the mice were observed over a 2‐week period for signs of Listeriosis.

For the acute immunity survival assays vitamin D‐deficient and vitamin D‐sufficient mice were inoculated with one LD_50_ dose of wild‐type *L. monocytogenes* 10403S. Additional mice were included as negative controls receiving diluent only (PBS).

For the adaptive immunity dose–response experiments, mice were immunized with ~1.0E+04 CFUs of the LADD vaccine strain. Additional mice in each group were immunized with PBS only. After 4 weeks, the immunized mice were divided into groups of five and challenged with a wild‐type *L. monocytogenes* dose. As an immunization control, PBS‐immunized mice were challenged with the lowest wild‐type *L. monocytogenes* challenging dose received by the immunized mice. A second PBS‐immunized control group was challenged with diluent only.

For the adaptive immunity survival assays, vitamin D‐deficient and vitamin D‐sufficient mice were immunized with ~1.0E+04 CFUs of the LADD vaccine strain. Ten additional vitamin D‐deficient and vitamin D‐sufficient mice were inoculated with PBS only. After 4 weeks, the immunized vitamin D‐deficient and vitamin D‐sufficient mice and five of the corresponding PBS‐immunized control mice were challenged with one LD_50_ dose of the wild‐type strain (~2.0E+06 CFUs). The remaining five PBS‐immunized mice were challenged with PBS only.

For all infection experiments, the mice were closely monitored over a 2‐week period and were immediately euthanized upon the onset of severe Listeriosis.

### Statistical analysis

The data collected from the dose–response experiments were utilized to calculate the LD_50_. For each dose–response experiment, the percent mortality versus the Listeria CFU dose was graphed. From these graphs, a linear or logarithmic regression equation was utilized to calculate the LD_50_. To determine if survival curves were statistically different from one another, data were analyzed using a log rank test.

Statistical analysis of blood serum calcium levels and 25(OH)D_3_ was performed using a Welch Two Sample *t*‐test.

All statistical analyses were done under the advisement of the UW‐Madison College of Agriculture and Life Sciences Statistical Consulting Group.

## Results

### Determination of the *Listeria monocytogenes* LD_50_ in C57Bl/6J mice

Survival assays were performed to study the effect of murine vitamin D deficiency on the acute immune response to a *L. monocytogenes* infection. In order to perform a survival assay, an accurate LD_50_ for Listeria was determined. Dose–response experiments were performed with the wild‐type strain 10403S on female C57Bl/6J mice infected via intravenous (i.v.) tail vein injection.

Once mice were confirmed vitamin D‐deficient and serum calcium levels determined to be no different than +D controls (Table 1), dose–response experiments were performed on two sets of mice, ages 15 and 16 weeks and ages 20 and 21 weeks. The LD_50_ for the 15‐week‐old vitamin D‐deficient mice was slightly higher than the 16‐week‐old vitamin D‐sufficient mice, while the opposite sensitivity was observed for the older set of mice (Table 2). With each set of mice, the calculated LD_50_ values were a factor of two different.

**Table 1:** Serum calcium and 25(OH)D_3_ levels in female C57Bl/6J mice

	Total serum Ca (mg/dL)		Serum 25(OH)D_3 _(ng/mL)	
	D‐deficient	D‐sufficient	D‐deficient	D‐sufficient
After vitamin D depletion	7.1 ± 0.2	8.9 ± 0.1*	ND	ND
At the time of infection	9.9 ± 0.1	10.0 ± 0.1	0†	22 ± 3*

* Statistically different from D‐deficient mice (p < 0.05).

^†^ Limit of detection is <4 ng/mL.

**Table 2:** *Listeria monocytogenes* 10403S LD_50_ determination for female C57Bl/6J mice

Listeria strain	Vitamin D status of mice	Age of mice (weeks)	No. of infected mice	Listeria dose range (CFUs)	LD50
wt	D‐deficient	15	25	3.32E+02–7.72E+04	4.71E+04
wt	D‐sufficient	16	56	5.80E+03–3.55E+05	7.70E+04
wt	D‐deficient	21	27	3.36E+04–8.39E+05	3.98E+05
wt	D‐sufficient	20	26	6.82E+04–1.16E+06	2.13E+05
LADD	D‐sufficient	17	48	1.22E+06–1.86E+09	1.55E+08
LADD	D‐sufficient	16	49	1.91E+07–4.50E+08	8.75E+07

**Figure 1:**
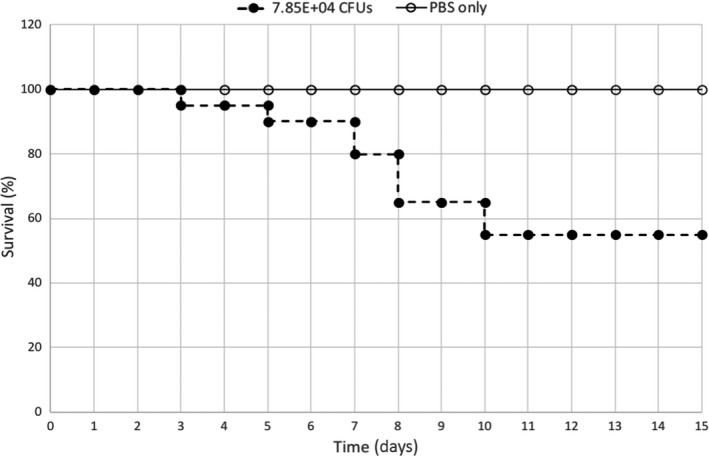
Confirmation of the *Listeria monocytogenes* 10403S LD_50_. Twenty vitamin D‐sufficient mice were infected with 7.85E+4 CFUs and five mice were dosed with an equivalent volume of PBS as a diluent control. Mouse mortality was monitored and scored over a 2‐week period. A statistically significant difference (*P* < 0.05) between the two survival curves was present.

To test the accuracy of the calculated LD_50_, a survival experiment was performed with a vitamin D‐sufficient population of 15‐week‐old female C57Bl/6J mice (Fig. 1). These data confirm that the LD_50_ determined in Table 1 for the 15 weeks vitamin D‐sufficient mice is ~7.85E+04 CFUs.

In addition to the wild‐type *L. monocytogenes* LD_50_ determination, the LD_50_ for the *Listeria monocytogenes* Δ*actA*Δ*inlB* vaccine strain, also known as the Listeria Attenuated Double Deletion (LADD) strain, was also determined (Table 2). Two LADD dose–response experiments were performed on vitamin D‐sufficient populations of female C57Bl/6J mice. The calculated LD_50_'s for these two experiments, 8.75E+07 and 1.55E+08 CFUs, were similar to a previously published report of 1.0E+08 CFUs (Brockstedt et al., 2004).

With an accurate *L. monocytogenes* LD_50_ established for C57Bl/6J mice, survival studies were then performed to assess the impact of vitamin D deficiency on the acute immune response to a *Listeria monocytogenes* infection.

### Vitamin D deficiency does not affect the murine acute immune response to a *Listeria monocytogenes* infection

**Figure 2:**
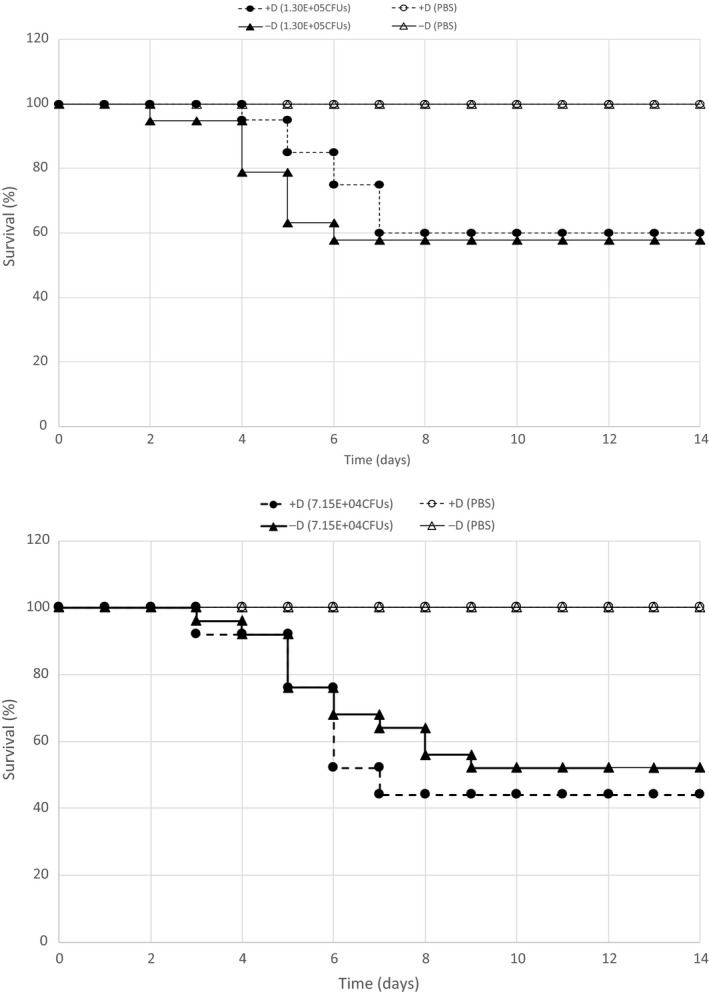
The effect of vitamin D status on the innate immune response to a Listeria infection. Two survival studies (top and bottom graph) were performed with two sets of mice with an age range of 16–17 weeks. In the top experiment, 20 vitamin D‐sufficient and 19 vitamin D‐deficient mice were infected with 1.30E+05 CFUs. As negative controls, five vitamin D‐sufficient and three vitamin D‐deficient mice were infected with diluent only (PBS). In the second experimental replicate (bottom), 25 vitamin D‐sufficient and 25 vitamin D‐deficient mice were infected with 7.15E+04 CFUs. As negative controls, five vitamin D‐sufficient and five vitamin D‐deficient mice were infected with diluent only (PBS). In each experiment, mortality was monitored and scored over a 2‐week period. No statistical difference in the survival curves of the D‐deficient mice compared to the D‐sufficient mice was found.

Two sets of age‐matched populations of mice, each containing a vitamin D‐deficient and vitamin D‐sufficient group, were infected with a *L. monocytogenes* 10403S LD_50_ dose and their mortality monitored and scored over a 2‐week period (Fig. 2). In the first set of mice, 57% of the vitamin D‐deficient and 60% of the vitamin D‐sufficient mouse populations survived the 2‐week experiment (Fig. 2). In a replicate, 52% of the vitamin D‐deficient and 44% of the vitamin D‐sufficient populations survived the infection (Fig. 2). In these studies, mortality began as early as 2 days after inoculation and the majority of the mice that were unable to fight the infection succumbed by day 7. Statistical analysis of the +D and −D survival curves for each experiment indicates there is no difference between the two infected populations. These data suggest the vitamin D status does not affect the acute immune response to a *L. monocytogenes* infection.

### Determination of the *Listeria monocytogenes* 10403S LD_50_ in LADD‐Immunized C57Bl/6J Mice

Like the acute immune response studies, survival assays were utilized to test the effect of vitamin D deficiency on the adaptive immune response to a *L. monocytogenes* infection. LADD‐immunized mice were predicted to have an increased resistance to a wild‐type *L. monocytogenes* challenge when compared to unimmunized mice. As a result, the LD_50_ of LADD‐immunized mice was determined.

A vitamin D‐deficient and vitamin D‐sufficient population of mice were immunized with 1.73E+04 CFUs of the LADD strain and given 4 weeks to develop immunity. A subsequent dose–response experiment was then performed with the wild‐type *Listeria monocytogenes* 10403S strain. The calculated LD_50_ for the vitamin D‐deficient and vitamin D‐sufficient immunized mouse populations, were 1.89E+06 CFUs and 4.89E+06 CFUs, respectively (Table 3). These data show that a LADD‐immunized population of mice, regardless of vitamin D status, has an increased resistance to the wild‐type *L. monocytogenes* strain.

**Table 3:** *Listeria monocytogenes* 10403S LD_50_ Determination for LADD‐Immunized C57Bl/6J mice

Vitamin D status of mice	Age of mice (weeks)	No. of infected mice	LADD immunization dose (CFUs)	WT Listeria dose range (CFUs)	LD50
D‐deficient	23	15	1.73E+04	6.53E+05–2.61E+06	1.89E+06
D‐sufficient	23	20		1.14E+06–5.65E+06	4.89E+06

With the *L. monocytogenes* LD_50_ established for LADD‐immunized mice, survival studies were performed to assess the impact of vitamin D deficiency on the adaptive immune response to a wild‐type *L. monocytogenes* infection.

### Vitamin D deficiency does not affect the murine adaptive immune response to a *Listeria monocytogenes* infection

**Figure 3:**
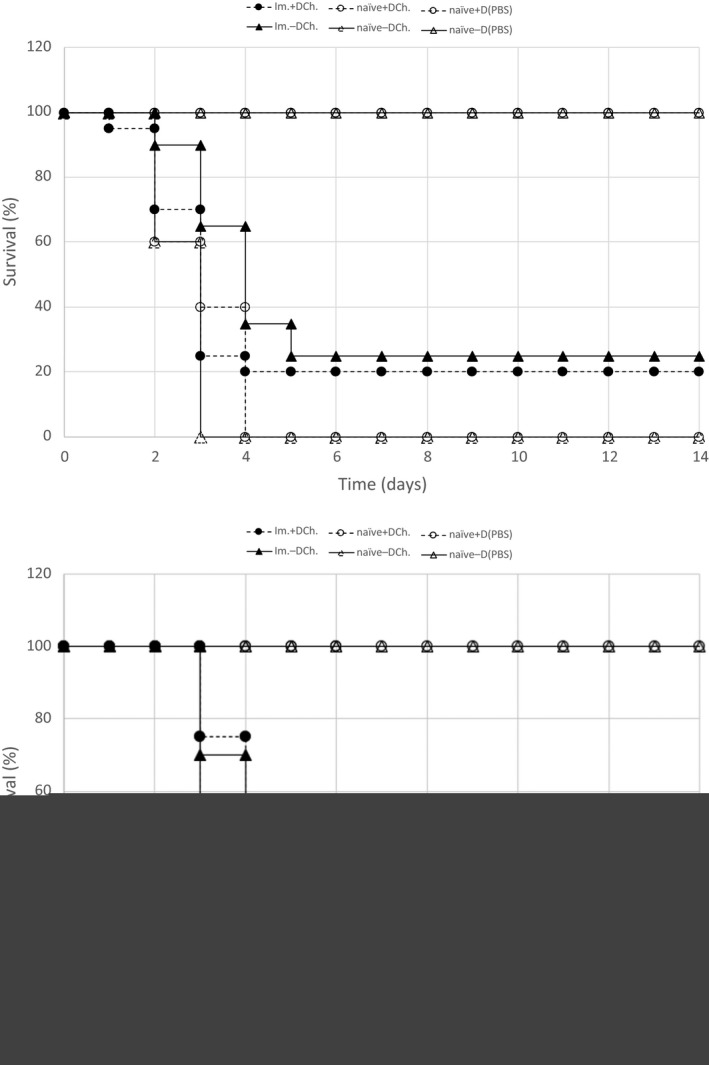
The effect of vitamin D status on the adaptive immune response to a Listeria infection. Two survival studies (top and bottom graph) were performed with two sets of mice with an age range of 20–21 weeks. In the top experiment, 20 vitamin D‐sufficient and 20 vitamin D‐deficient mice were immunized with the LADD strain (1.23E+04 CFUs). As an immunization and diluent‐only control, 10 mice were immunized with diluent only (PBS). After 30 days, the 20 immunized mice (Im. +D Ch. and Im. −D Ch.) and five PBS‐immunized mice (naïve +D Ch. and naïve −D Ch.) from each group were challenged with an ~1 LD_50_ dose (4.16E+06 CFU) of wild‐type *L. monocytogenes* 10403S strain. The remaining five PBS‐immunized control mice [naïve −D (PBS) and +D naïve (PBS)] were rechallenged with diluent only (PBS). In the second experimental replicate (bottom), 25 vitamin D‐sufficient and 25 vitamin D‐deficient mice were immunized with the LADD strain (1.43E+04 CFUs). Ten mice were immunized with diluent only (PBS). After 30 days, the 25 immunized mice (Im. +D Ch. and Im. −D Ch.) and five PBS‐immunized mice (naïve +D Ch. and naïve−D Ch.) from each group were challenged with an ~LD_50_ dose (3.22E+06 CFU) of wild‐type Listeria. The remaining five PBS‐immunized control mice [naïve −D (PBS)and +D naïve (PBS)] were rechallenged with diluent only (PBS). In each experiment, mortality was monitored and scored over a 2‐week period. Statistical analysis revealed no significant difference in the survival curves of the D‐deficient mice compared to the D‐sufficient mice.

To investigate the adaptive immune response to a *Listeria monocytogenes* infection, listeriosis survival studies were performed on two sets of mice each containing a vitamin D‐deficient and vitamin D‐sufficient population of mice. These mice were LADD‐immunized with ~1.0E+04 CFUs and then challenged with the ~one LD_50_ dose of the wild‐type strain that was determined for immunized mice. In the first experiment, 25% of the vitamin D‐deficient mice and 20% of the D‐sufficient mice survived the 2‐week infection. In the second experimental replicate, 35% of both the D‐deficient and D‐sufficient populations survived the 2‐week experiment (Fig. 3).

These survival studies show a LADD‐immunized vitamin D‐deficient mouse population has a statistically similar Listeriosis survival rate as a LADD‐immunized vitamin D‐sufficient mouse population. These data demonstrate that vitamin D status does not affect the murine adaptive immune response to a *L. monocytogenes* infection.

## Discussion

A mouse model of vitamin D deficiency was utilized to study the effect of vitamin D status on the mortality in response to an infection with a virulent strain of *Listeria monocytogenes*. Dose–response experiments were performed with the wild‐type *Listeria monocytogenes* strain 10403S on unimmunized wild‐type mice to determine the LD_50_ for both vitamin D‐deficient and vitamin D‐sufficient mouse populations (Table 2). The LD_50_ determined in this study, for both the vitamin D‐deficient and vitamin D‐sufficient populations, are similar to the previously reported LD_50_ (1.0E+05 CFUs) for this strain (Bishop and Hinrichs, 1987; Mainou‐Fowler et al., 1988; Portnoy et al., 1988) (Table 2). In addition, the survival studies showed no difference in the mortality between the vitamin D‐deficient and vitamin D‐sufficient mouse populations (Fig. 2). These data indicate that vitamin D status has no effect on the acute immune response to a Listeria infection which could involve both innate and adaptive immunity. Because of the short duration, as early as 2 days, in which naïve mice inoculated with Listeria died, it is likely that components of the innate immune system were employed. But due to the lag in mortality, as late as 9 or 10 days in some mice, it is possible that both innate and adaptive immune systems were activated in these studies.

The effect of vitamin D deficiency on the adaptive immune system was also analyzed. Due to an increased resistance because of the Listeria immunization, a new LADD‐immunized LD_50_ was determined for both vitamin D‐deficient and vitamin D‐sufficient mice (Table 3). These data show that the LADD immunization results in an increased resistance for both the vitamin D‐deficient and vitamin D‐sufficient populations. A LADD immunization dose of ~1.0E+04 CFUs resulted in a ~10‐fold increase in resistance to a wild‐type Listeria challenge in both the vitamin D‐deficient and the vitamin D‐sufficient mice as compared to the LD_50_ for naïve populations of mice with the same vitamin D status (Tables 2 and 3).

Survival assays performed on two age‐matched LADD‐immunized sets of mice, each set containing a vitamin D‐deficient and vitamin D‐sufficient mouse population, showed no difference in rates of mortality from a Listeria infection (Fig. 3). These data demonstrate that vitamin D status does not affect the adaptive immune response to a Listeria infection.

The idea that vitamin D and/or its active metabolite plays some role in immunity is based predominantly on *in vitro* studies. *In vivo* studies on the effect of vitamin D deficiency on immunity, apart from autoimmunity, are sparse and those in existence do not provide a consistent picture for the role of vitamin D. For example, Ehrchen et al. (2007) reported VDR knockout mice are less susceptible to Leishmania major infection compared to wild‐type controls. In contrast, Hertting et al. (Hertting et al., 2017) reported that vitamin D‐deficient mice are more susceptible to infection by *E. coli* strain CFT073.

A number of clinical studies have been reported on the effect of vitamin D on infections. Five meta‐analyses of primary trials have indicated that vitamin D has a protective effect (Charan et al., 2012; Bergman et al., 2013) while three reported no statistical effect of vitamin D supplementation (Mao and Huang, 2013; Xiao et al., 2015; Vulchard et al., 2016). Although there are several possible reasons for these disparate results, conclusions of whether vitamin D plays a role in immunity cannot be reached on the basis of these clinical observations. In our study, the absence of 25(OH)D in blood and achievement of severe hypocalcemia clearly establishes vitamin D deficiency. Because hypocalcemia itself irrespective of vitamin D deficiency may have profound biological effects, we kept the animals vitamin D‐deficient but corrected the hypocalcemia by providing the same diet but containing high calcium plus lactose that enhances calcium absorption by a non‐vitamin D‐dependent mechanism. Under these circumstances, the effect of vitamin D deficiency itself independent of hypocalcemia can be studied.

The results from this study indicate that both innate and acquired immunities are not vitamin D‐dependent. These results are consistent with the study done in VDR knockout mice where both old and young mice were able to clear primary and secondary infections of *Listeria* (Bruce et al., 2009). Because our study was confined to *Listeria* infection in mice further studies with different infections and hosts are needed to establish firmly the idea that vitamin D does not play a role in both innate and acquired immunity.
